# Antimicrobial Peptides from Marine Proteobacteria

**DOI:** 10.3390/md11103632

**Published:** 2013-09-30

**Authors:** Florie Desriac, Camille Jégou, Eric Balnois, Benjamin Brillet, Patrick Le Chevalier, Yannick Fleury

**Affiliations:** University of Brest, LUBEM EA 3882, SFR 148, Quimper 29000, France; E-Mails: florie.desriac@univ-brest.fr (F.D.); camille.jegou@univ-brest.fr (C.J.); eric.balnois@univ-brest.fr (E.B.); benjamin.brillet@univ-brest.fr (B.B.); patrick.lechevalier@univ-brest.fr (P.L.C.)

**Keywords:** Proteobacteria, marine, peptide, antimicrobial, antibiotic, antifungal, NRPS

## Abstract

After years of inadequate use and the emergence of multidrug resistant (MDR) strains, the efficiency of “classical” antibiotics has decreased significantly. New drugs to fight MDR strains are urgently needed. Bacteria hold much promise as a source of unusual bioactive metabolites. However, the potential of marine bacteria, except for Actinomycetes and Cyanobacteria, has been largely underexplored. In the past two decades, the structures of several antimicrobial compounds have been elucidated in marine Proteobacteria. Of these compounds, polyketides (PKs), synthesised by condensation of malonyl-coenzyme A and/or acetyl-coenzyme A, and non-ribosomal peptides (NRPs), obtained through the linkage of (unusual) amino acids, have recently generated particular interest. NRPs are good examples of naturally modified peptides. Here, we review and compile the data on the antimicrobial peptides isolated from marine Proteobacteria, especially NRPs.

## 1. Introduction

Since the beginning of antibiotic use, bacterial resistance has been a main public health issue. Despite the need for new active compounds, only a few new antibacterial structure classes have been approved for human use in the past 40 years. For example, the lipopeptide antibiotic daptomycin was approved in 2003, and it was the first natural product-based antibiotic from a new structure class to be launched onto the market in this time period. Natural sources provide the most diverse compounds for drug development [1]. Among marine organisms, bacterioplanktons should be now regarded as a valuable reserve of original molecules. However, the discovery of bioactive compounds from marine bacterioplankton often depends on the ability to culture the microorganisms [2], because cultivation would probably be necessary to obtain a regular supply of bacterioplankton-derived compounds at a commercial scale [3]. Even though great improvements have been made, new culture media still need to be developed to ensure sustainable cultures of the microorganisms of interest [4].

The enhancement of genomic tools has provided further insight into marine biodiversity [5,6,7]. The most abundant bacteria tend to be α-Proteobacteria representatives, whereas those successfully cultured are generally members of the γ*-*Proteobacteria class [2]. The phylum Proteobacteria consists of Gram-negative bacteria with diverse lifestyles that inhabit various environments; the name of the phylum was first proposed by Stackebrandt *et al.* [8] for the group of “purple bacteria and their relatives” and the clade includes the well-known α-, β-, γ-, δ- and ε-Proteobacteria classes (Table 1). *Vibrio*, *Pseudoalteromonas* and *Pseudomonas* are some well-known proteobacterial genera frequently observed in marine environments. Recently, new species have been discovered and led to the creation of the *Candidatus* ζ-Proteobacteria class [9].

**Table 1:** Current classification of Proteobacteria.

Phylum Proteobacteria					
**Class**	**α-Proteobacteria**		**Class**	**β-Proteobacteria**	
Orders	Caulobacterales	Rhizobiales	Orders	Burkholderiales	Neisseriales
	Kiloniellales	Rhodobacterales		Ferritrophicales	Nitrosomonadales
	Kopriimonadales	Rhodospirillales		Gallionellales	Procabacteriales
	Kordiimonadales	Rickettsiales		Hydrogenophilales	Rhodocyclales
	Magnetococcales	Sneathiellales		Methylophilales	Unclassified taxa
	Parvularculales	Sphingomonadales			
	Unclassified taxa				
**Class**	**γ-Proteobacteria**		**Class**	**δ-Proteobacteria**	
Orders	Acidithiobacillales	Orbales	Orders	Bdellovibrionales	Desulfuromonadales
	Aeromonadales	Pasteurellales		Desulfarculales	Myxococcales
	Alteromonadales	Pseudomonadales		Desulfobacterales	Syntrophobacterales
	Cardiobacteriales	Salinisphaerales		Desulfovibrionales	Unclassified taxa
	Chromatiales	Thiotrichales		Desulfurellales	
	Enterobacteriales	Vibrionales			
	Legionellales	Xanthomonadales			
	Methylococcales	Unclassified taxa			
	Oceanospirillales				
**Class**	**ε-Proteobacteria**		**Class**	**ζ-Proteobacteria (candidatus)**	
Orders	Campylobacterales		Order	Mariprofundales	
	Nautiliales				
	Unclassified taxa				

Even though Proteobacteria is the most abundant phylum in marine environments (abundance between 50% and 80%), only a few bioactive compounds have been described from those microorganisms [10]. In contrast, many natural products have been isolated from the phylum Actinobacteria, which accounts for only 5% to 10% of total bacterioplankton [5,6,7,11]. Several kinds of natural products from marine microorganisms are reported in the literature. Many of them are peptides, polyketides or hybrids thereof [12].

Antimicrobial peptides produced by bacteria can be classified according to how they are synthesised: Via the ribosomal (bacteriocin) or the non-ribosomal pathway. To date, no marine bacteriocins have been characterised from marine Proteobacteria. Nonribosomal peptide synthetase (NRPS) and polyketide synthase (PKS) are the hallmarks of secondary metabolites. These biosynthetic pathways have been extensively studied regarding their ability to generate a wide variety of compounds presenting antimicrobial, siderophore, antitumour, surfactant, immunomodulatory, or toxic properties [13]. Since they were first described in the 1970s [14,15], NRPS systems have been widely studied. In addition, recent advances in genomics have led to precise identification of NRPS genes. The biological activity of nonribosomal peptides (NRPs) is probably enhanced by the presence of non-proteinogenic amino acids, as demonstrated for some peptides [16]. Unlike ribosomal peptide synthesis, NRPS enzymes are able to select and incorporate more than 100 amino acids. Furthermore, these amino acids can even be modified by NRPS itself, hence increasing the diversity of the generated molecules. As a consequence, NRPs are an important source of novel bioactive molecules.

This review gives an overview of antibiotic peptides produced by NRPSs in the most abundant marine bacteria phylum, Proteobacteria. Here, we describe the structure, antimicrobial activity, structure-activity relationships (when available) and finally biosynthesis mechanisms (when elucidated) of marine NRPs. When the biosynthesis has only been elucidated in another bacterium (e.g., in a non-marine bacterium), it is given in the text and the information is mentioned. The comparison of NRP antimicrobial activity remains difficult due to the great diversity and non-standardised protocols that are used in the literature: Microbial strains, target cell concentrations (10^4^ to 10^8^ CFU mL^−1^), physiological states, incubation times or temperatures, culture media, liquid or diffusion assays, *etc*. all vary among studies. Marine antimicrobial peptides will be described according the systematic classification of their bacterial sources (α-, β-, γ-, ε-Proteobacteria), as indicated in Table 2.

## 2. Overview of Nonribosomal Peptide Synthetases (NRPSs)

NRPSs are multimodular enzymes. Each module is divided into domains. Three domains are required for synthesis of the peptide backbone: The adenylation (A) domain, the peptidyl carrier protein (PCP) and the condensation (C) domains [17]. NRPSs have already been presented and discussed in other reviews [13,17,18,19,20].

**Table 2:** Antimicrobial peptides isolated from marine Proteobacteria*.*

Class/Order	Producers	Compounds	Activity	Biosynthesis
**α-Proteobacteria**				
Rhodobacterales	*Oceanibulbus indolifex*	Cyclic dipeptides	Antibacterial	Unknown
	*Phaeobacter* sp.	Indigoidine	Antibacterial (Gram-negative)	NRPS
**γ-Proteobacteria**				
Vibrionales	*Vibrio* sp.	Andrimid Moiramide (pseudopeptides)	Antibacterial	Hybrid NRPS-PKS (<100 kDa)
		Kahalalides (depsipeptides)	Antifungal	Unknown
	*Photobacterium halotolerans*	Holomycin (pyrrothine)	Bacteriostatic	NRPS described in *Streptomyces clavuligerus*
	*Photobacterium* sp.	Unnarmicins (depsipeptides)	Antibacterial (Gram-negative)	Unknown
		Ngercheumicins (depsipeptides)	Antibacterial (Gram-negative)	Unknown
		Solonamides (depsipeptides)	Antibacterial (Gram-negative)	Unknown
Alteromonadales	*Pseudoalteromonas* sp.	Thiomarinols (pyrrothine)	Antibacterial	NRPS (pTLM1)
		Cyclopeptides	Antibacterial	Unknown
		Lipopeptides	Antibacterial	NRPS
Pseudomonadales	*Pseudomonas* sp*.*	Massetolides (cyclic lipopeptides)	Antimycobacterial	NRPS described in *Pseudomonas fluorescens*
**δ-Proteobacteria**				
Myxococcales	*Myxococcus fulvus*	Myxovalargins (polyketide/polypeptide hybrid)	Antibacterial	Unknown
		Althiomycin (polyketide/polypeptide hybrid)	Antibacterial	Hybrid NRPS-PKS described in *Mycococcus xanthus*
		Myxothiazols	Antifungal	NRPS described in *Stigmatella aurantiaca*
	*Paraliomyxa miuraensis*	Miuraenamides (depsipeptides)	Antifungal	Unknown

**Figure 1:**
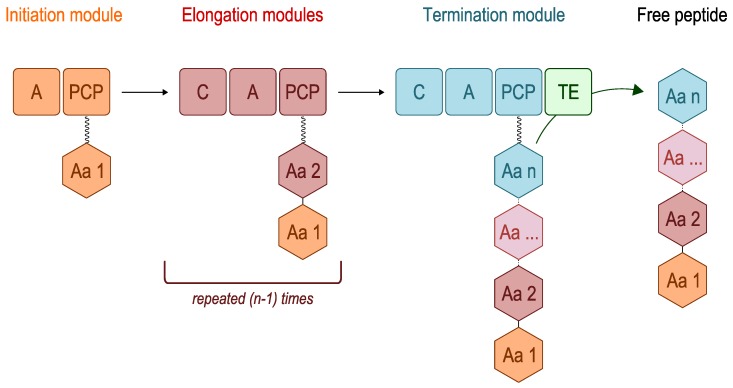
Diagram of NRP biosynthesis. Aa: amino acid; A: adenylation domain; C: condensation domain; PCP: peptidyl carrier protein; and TE: thioesterase domain.

Many NRPs are synthesised according to the colinearity rule (Figure 1). The number and order of modules in the genome represent the number and order of amino acid residues in the final product [21]. Although NRPS enzymes have nothing in common with aminoacyl-tRNA-synthetase from a structural point of view, A domains have similar functions; they control amino acid substrate activation, and their simultaneous activation as aminoacyl adenylates is at the expense of ATP. This domain is composed of about 550 amino acid residues, among which about 10 residues are used to predict substrate specificity [17]. The PCP domain, also known as the thiolation domain (T), is composed of a segment of 80–100 amino acids long and represents the NRPS transport unit. The activated substrate is transferred to the thiol group of the 4′-phosphopantetheine on the PCP and covalently bound through a thioester linkage [19,22]. Once the aminoacyl substrate has undergone all necessary modifications, the C domain (about 450 amino acid residues long) catalyses the formation of a C–N bond between the two aminoacyl substrates bound to modules adjacent to the PCP domain [19,23]. The first A domain and PCP initiate NRP biosynthesis, then the C, A and PCP domains are repeated several times, depending on the number of amino acids that compose the final NRP product. During synthesis, all intermediates remain covalently bound to the enzyme complex [18]. A thioesterase (TE) terminal domain is frequently involved in the release of the peptide product. This enzyme of 250 amino acids in length is only found in the termination module of the NRPS. The peptide can also be released through the action of a reductase (R) domain that converts the thioester into an alcohol, for example.

Furthermore, the NRPS system uses additional domains involved in NRP tailoring, e.g., the epimerisation (E), heterocyclisation (Cy), oxidation (Ox), methylation (M) and formylation (F) domains [24]. All these tailoring domains contribute to NRP structural diversity. The potential of NRPs in drug development is highly promising; therefore, diverse strategies have been developed to screen for them.

The linear NRPS model is the best known, but two other models have also been described. In the iterative model, the same domains or modules are used more than once for the synthesis of one NRP. In the non-linear model, domain organisation may differ from the classical one (C-A-PCP) described above. The hybrid NRPS-PKS system belongs to this latter model. All three models and the diversity of assembly lines have been well described in the literature [25]. Iterative and non-linear strategies can complicate predictions based on the primary structure of the enzyme and thereby limit NRP prediction.

### 2.1. Genome-Based Strategy

With the progress in DNA sequencing technology, genomic sequences have been determined for a large number of species. According to the Genome Online Database (GOLD), 6330 complete bacterial genomes are now available and 14,986 are about to be published. Bioinformatics tools have undergone rapid improvement that has led to “genome mining” approaches. Most studies have focused on NRPS, PKS, or hybrid NRPS-PKS pathways, because the large size and repeating motifs in their genomic nuclear sequences make them relatively simple to identify and analyse [26]. Although the Basic Local Alignment Search Tool (BLAST) is widely used for genome mining and NRPS discovery, specific NRPS-PKS bioinformatics tools have been developed; for more details on the software available for NRPS-PKS analyses, see [27]. Since this software review was published, two freely available online applications, NapDos and antiSMASH, have been introduced [28,29]. Most of the genome-based reports dealing with the discovery of NRPS genes have used targeted gene inactivation as a proof of NRP biosynthesis because the metabolites are constitutively produced [30,31,32]. 

### 2.2. Proteomics Strategy

Given that genomes are not always available and that NRPS assembly lines have been discovered without following colinearity rules [21], alternative methods have been developed. One of these methods is named PrISM (Proteomic Investigation of Secondary Metabolism) [33], and uses a strategy based on the large size of NRPS enzymes (often more than 2000 amino acid residues). The workflow consists of first performing SDS-PAGE electrophoresis of the soluble proteome to excise the putative NRPS (protein of about 225 kDa). After trypsin digestion, the peptides are analysed and then degenerate primers are designed. Gene cluster sequencing and identification leads to possible hypothetical structures for the targeted molecules and hence, the development of a relevant purification protocol. One aspect of this integrated approach is detecting peptide fragments containing phosphopantetheinyl, the cofactor involved in all PCP domains. Phosphopantetheinyl was shown to be linked to the Ser residue via a phosphodiester linkage that is labile in tandem mass spectrometry (MS/MS) [34]. This PrISM strategy has been used on a collection of 22 environmental isolates, for which no genomic information was available, and led to the identification of six related lipoheptapeptides [33]. 

## 3. Antimicrobial NRPs from Marine Proteobacteria

Numerous published studies have been devoted to the characterisation of marine bacterial molecules. However, far fewer studies have focused on antimicrobial peptides from Proteobacteria. In this review, we will mainly describe molecules from the α-, γ- and δ-Proteobacteria classes. There are not yet any reports of molecules from β-, ε- and ζ-Proteobacteria. To complete this list of molecules, antimicrobial peptides isolated from marine animals, and for which a proteobacterial origin is assumed, will also be presented. Finally, we will not describe all known genera of Proteobacteria, because, within a given class, the available data on antimicrobial peptides involve only a few taxa.

### 3.1. α-Proteobacteria

#### 3.1.1. Cyclic Dipeptides

A peptide compound with antibiotic activity has been isolated from *Oceanibulbus indolifex* [35]. This species produces three cyclic dipeptides: *cyclo*-(Phe-Pro), *cyclo*-(Tyr-Pro) and *cyclo*-(Leu-Pro). Using solid phase peptide synthesis (SPPS), it has been shown that the two first dipeptides have strong antibiotic activity with micromolar minimum inhibitory concentration (MIC) values [36]. No biosynthesis pathways have been described for these cyclodipeptides in this species.

#### 3.1.2. Indigoidine

A blue pigment has been shown to be associated with the ability of *Phaeobacter* sp. Y4I to inhibit *Vibrio fischeri* [37]. This pigment, referred to as indigoidine, is produced by condensation of two glutamine residues via a NRPS-based biosynthetic pathway. Indigoidine biosynthesis genes are *igiB*, coding for a 6-phosphogluconate dehydrogenase, *igiC*, for a glutamate racemase and *igiD* for indigoidine synthase. This gene, also known as *indC*, has been identified in a phylogenetically diverse group of microorganisms, including α-, β- and γ-Proteobacteria, as well as several *Streptomyces* species [38].

### 3.2. γ-Proteobacteria

#### 3.2.1. Andrimid and Moiramide

**Figure 2:**
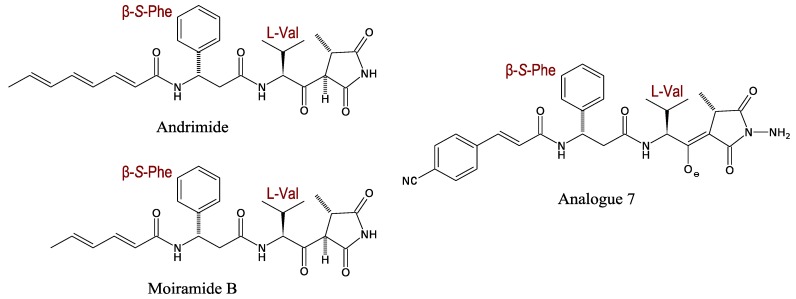
Structures of andrimid, moiramide B and their analogue 7.

Andrimid and its analogue moiramide are probably the most studied pseudopeptide antibiotics (Figure 2). Andrimid was first described in *Vibrio* in 1994, and shown to be produced by a *Vibrio* bacterium found in a marine sponge (*Hyatella* sp.) [39]. These antibiotics seem to be widespread in the γ-Proteobacteria as several *Vibrio* species [39,40,41], the marine *Pseudomonas fluorescens* [42], *Pantoea agglomerans* [43] and a symbiotic planthopper *Enterobacter* [44] have been shown to produce them.

Andrimid consists of four components: (1) An unsaturated fatty-acid chain; (2) A β-Phe moiety; (3) A l-Val derived β-ketoamide moiety and (4) A pyrrolidinedione. Andrimid and moiramide differ only in the length of their fatty acid chains. A broad antibacterial spectrum has been described for andrimid with activity towards Gram-positive and -negative bacteria [42]. The broad antibacterial activity is likely linked to the molecular target of these compounds, *i.e.*, the widely conserved acetyl-CoA carboxylase (ACC) [45].

Using chemical synthesis, Pohlmann *et al.* showed that a pyrrolidinedione head is involved in antimicrobial activity and that the fatty acid chain is especially involved in cell penetration [46]. In 2006, the same research team focused on the two internal parts of moiramide, *i.e*., the β-(*S*)-Phe and the l-Val derived β-ketoamide [47]. The substitution of Phe by a non-aromatic amino acid leads to the loss of antibacterial activity. A more lipophilic substitution on the phenyl ring of the β-(*S*)-Phe improves antibacterial activity, suggesting that this moiety is involved in the cell penetration of the molecule, as well as the fatty acid chain. An isopropyl chain modification of the l-Val-derived β-ketoamide enhances antibacterial activity against Gram-positive bacteria. Finally, a new derivative, named analogue 7, has been synthesised to obtain a more active derivative and better solubility in water for therapeutic use (Figure 2).

**Figure 3:**
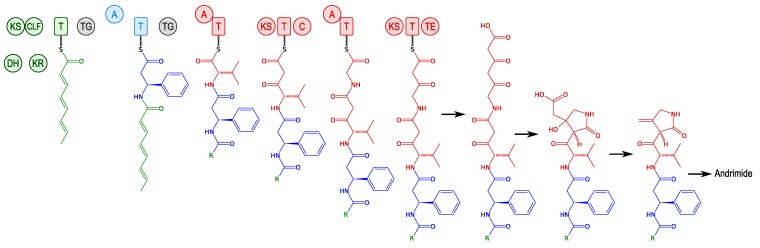
The NRPS-PKS synthesis of andrimid [43]. KS: ketosynthase; DH: dehydratase; CLF: chain length factor; KR: ketoreductase; T: thiolation domain; TG: transglutaminase; A: adenylation domain; C: condensation domain; TE: thioesterase.

The putative hybrid NRPS-PKS system involved in andrimid synthesis is a good example for studying the hybrid mechanism of synthesis because the enzyme is relatively small (less than 100 kDa [48]). The gene cluster comprises four recognisable sections that play distinct roles: (1) Formation of a polyunsaturated fatty acid by an iterative type II PKS; (2) Formation and insertion of β-Phe; (3) Construction of a succinimide precursor from Val, Gly, and C2 units from two equivalents of malonyl-CoA; and (4) Resistance and enzyme priming (Figure 3) [43,49].

An original approach for constructing derivatives was used by Evans *et al*. [48]. They created a new library of mutants with thiotemplates altered by targeted mutations on the Val substrate binding site. Hundreds of mutants, *i.e*., hundreds of strains with enzymatic variations and the unusual molecules putatively generated thereof, were screened using mass spectrometry according to their structure. This orthogonal approach highlights the potential of combinatorial biosynthesis as an alternative to the chemical synthesis of complex natural compounds, with the similar efficiency and even improved biological activity.

#### 3.2.2. Kahalalides

**Figure 4:**
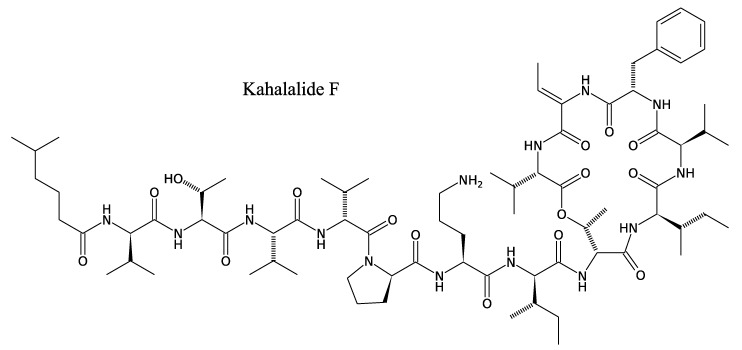
Structure of kahalalide F.

Other well-characterised, unusual antibiotic peptides are the kahalalides, a family of depsipeptides with variable size and peptide series, ranging from C31 (tripeptide) to C77 (tridecapeptide) and carrying different fatty acid chains [50]. These compounds, first isolated from the herbivorous marine mollusc *Elysia* and its algal food source *Bryopsis pennata*, have also been described in some *Vibrio* sp. strains isolated from the same mollusc [51]. Although the origin of kahalalides remains uncertain, Hill *et al*. suggest that *Elysia rufescens* acquires kahalalide-producing microbes from the surface of *Bryopsis* and then retains these microbes as symbionts. This family of depsipeptides has been reviewed [50]; therefore, we present here only the main characteristics of these peptides. Kahalalides and especially kahalalide F (Figure 4) are known for their antifungal and antitumour activities. Phase II clinical trials are underway in regard to the latter.

These compounds are active against several fungi such as *Candida albicans*, *Cryptococcus neoformans*, *Mycobacterium intracellulare* and *Aspergillus fumigates* [52]. Their chemical synthesis has made it possible to characterise structure-activity relationships and has resulted in a derivative with increased biological activity. The genes that govern kahalalide synthesis have not yet been characterised, perhaps due to the unknown origin of kahalalide-related depsipeptides. NRPS genes are most likely to be involved in kahalalide biosynthesis, as suggested by the structure of these molecules (Figure 4). Other *Vibrio* genomes are currently under investigation, especially *Vibrio shilonii* (Gi01413-GOLD), a species closely related to the kahalalide F-producing strain. Therefore, kahalalide biosynthesis pathways may be brought to light in the near future.

#### 3.2.3. Holomycin

**Figure 5:**
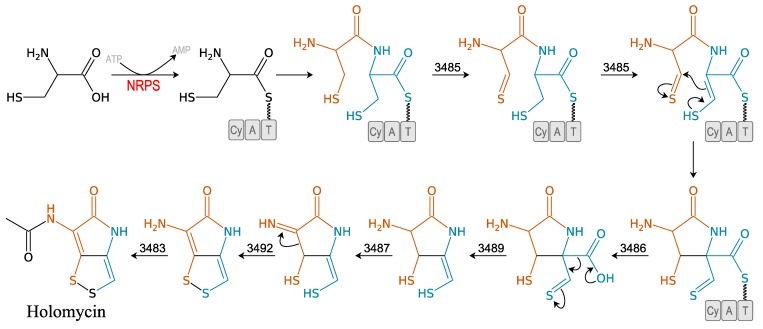
Biosynthesis of holomycin [31]. 3485: acyl-CoA dehydrogenase; 3486: thioesterase domain; 3489: phosphopantothenoylcysteine decarboxylase; 3487: glucose-mathanol-choline oxydoreductase; 3492: thioredoxin-disulphide reductase; 3483: acetyltransferase; Cy: cyclisation domain; A: adenylation domain; T: thiolation domain or peptidyl carrier protein (PCP).

Recently described, holomycin is produced by the *Photobacterium halotolerans* S2753 [41]. It is the final product of a Cys-Cys dipeptide precursor that displays a broad spectrum of antibacterial activity [31]. This pyrrothine compound is an excellent example of how natural amino acid residues can be modified to form “exotic” molecules (Figure 5). Prior to this study, this pyrrothine antibiotic compound had only been isolated from Gram-positive bacteria and especially from the actinomycete *Streptomyces clavuligerus* [53]. Holomycin exerts a bacteriostatic effect on both Gram-negative and -positive bacteria species [54]. It causes rapid inhibition of RNA chain elongation [54,55]. The gene cluster responsible for its synthesis in *S. clavuligerus* is a one-module NRPS system (Figure 5) [31].

Surprisingly, until 2010, the production of holomycin had only been demonstrated in Gram-positive bacteria [41]. Horizontal gene transfer is the most likely explanation for its occurrence in Gram-negative heterotrophic bacteria. A high number of mobile genetic elements have been reported in this family [56,57].

#### 3.2.4. Unnarmicins

**Figure 6:**
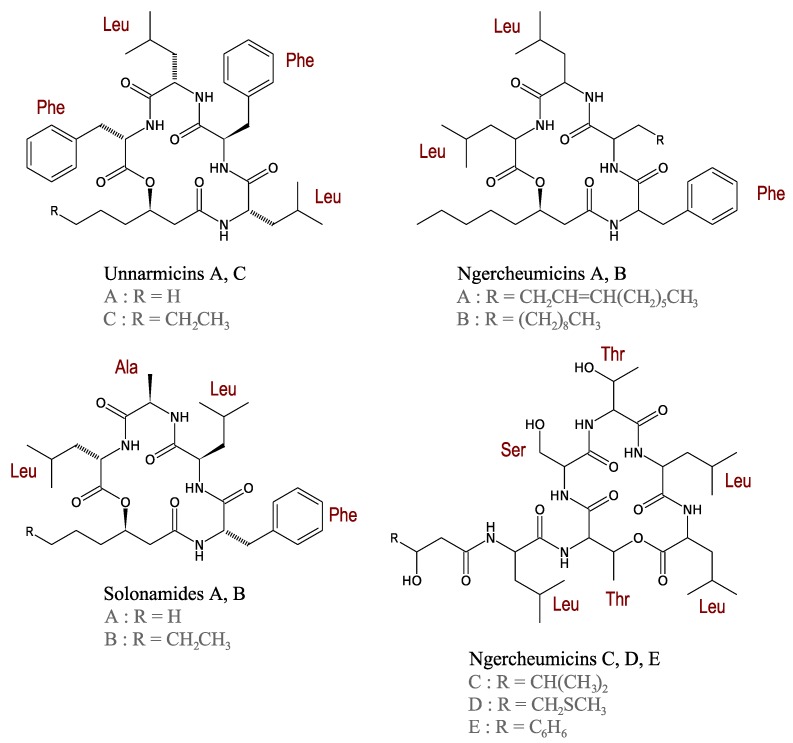
Structures of unnarmicins, ngercheumicins and solonamides.

Unnarmicins are depsipeptides isolated from the fermentation broth of a marine *Photobacterium* sp. MBIC06485. Unnarmicins contain two Leu, two Phe, and one 3-hydroxyoctanoyl moiety or one 3-hydroxyhexanoyl group in unnarmicins A and C, respectively (Figure 6). These compounds exert an antibacterial activity only towards species of the *Pseudovibrio* genus, one of the most common α-Proteobacteria genera in the marine environment [58].

Although the *Pseudovibrio* genus has not been shown to be pathogenic, unnarmicins can be included in culture media, along with other selective marine antibiotics such as korormicin [59], to repress fast-growing bacteria and thus facilitate the isolation and purification of slow-growing bacteria from marine environments.

#### 3.2.5. Ngercheumicins

Other original depsipeptides from *Photobacterium* strains that are active against the non-pathogenic *Pseudovibrio denitrificans* were patented in 2007: The ngercheumicins [60]. To date, five depsipeptides named ngercheumicins A–E have been purified and characterised (Figure 6). Ngercheumicins A and B have a depsipeptide macrocycle containing one Phe and two Leu residues with different fatty acid tails. Ngercheumicins B–D have a macrocycle composed of three Leu, two Thr and one Ser with no fatty acid tail (Figure 6). Although these compounds only target bacteria for which no pathogenesis has been described to date, as noted for unnarmicins, ngercheumicins can be added to culture media to promote slowly-growing marine bacteria. 

#### 3.2.6. Solonamides

Another marine *Photobacterium* produces cyclodepsipeptides called solonamides. The description in this genus of various depsipeptides suggests that this type of compound may be a common feature in *Photobacterium*. In terms of structure analogy, solonamides are closely related to unnarmicins and ngercheumicins (Figure 6), with a macrocycle containing one l-Phe, one l-Ala, one l-Leu and one d-Leu residue with a hydroxyoctanoyl or a hydroxyhexanoyl moiety in solonamides B and A, respectively [61]. In the same study, no antibacterial activity has been observed for solonamides A and B however the activity has only been assessed against *Vibrio anguillarum* and *S. aureus.* Nevertheless, solonamide B has been reported to reduce the expression of both *hla* and *rnaIII* of methicillin-resistant *Staphylococcus aureus* (MRSA), two genes involved in strain virulence controlled by an *agr*-dependent *quorum sensing* system. The structural similarity of the solonamides and the auto-inducing peptides involved in *S. aureus* quorum sensing suggests that solonamides may be competitive inhibitors of the *agr* system [61]. The reduced activity of solonamide A compared with solonamide B indicates that the overall hydrophobicity of the depsipeptides due to the fatty acid chain may have an important impact on the inhibition of expression of the virulence gene in *S. aureus.* As the importance of quorum sensing in the development of virulence in pathogenic bacteria has become clear, compounds that disrupt quorum sensing alone [62] or in combination with classical antibiotics [63] have been proposed as a new anti-infective therapy. However, this strategy remains disputed [64], therefore requiring further investigation. 

To our knowledge no data are available on biosynthetic pathways of cyclodepsipeptides (unnarmicins, ngercheumicins and solonamides). Nonetheless, the structural features of these cyclodepsipeptides suggest that they are synthesised on NRPS machinery.

#### 3.2.7. Thiomarinols

**Figure 7:**
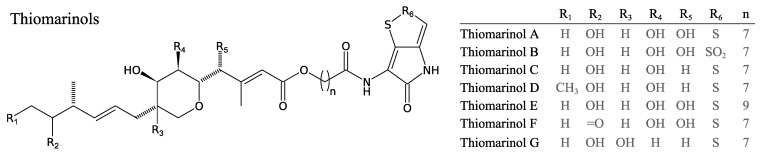
Structure of thiomarinols produced by *Pseudoalteromonas* sp. SANK 73390.

A well-characterised hybrid NRPS-PKS system is responsible for the biosynthesis of thiomarinol compounds in *Pseudoalteromonas* sp. SANK 73390. These compounds, related to the holomycin antibiotics discussed above, are hybrids of two antibiotics: A pseudomonic acid derivative (marinolic acid) and pyrrothine, linked via an ester bond [65]. Thiomarinols have potent activity against both Gram-negative and -positive bacteria, especially against MRSA. Liquid-liquid extraction of the cell-free supernatant has shown that *Pseudoalteromonas* sp. SANK 73390 produces seven thiomarinol analogues (Figure 7) [65,66,67]. Total DNA sequencing of the producer strains and mutagenesis have revealed that the thiomarinol biosynthesis system is carried by a plasmid designated as pTML1 [68]. pTML1 also carries 21 open-reading frames (ORFs) that encode PKS genes involved in the synthesis of marinolic acid [68]. Mutagenesis in PKS or NRPS has shown that marinolic acid and pyrrothine can be produced separately. When defective mutants in either pathway are co-fermented, thiomarinol production is restored, indicating that an external product (marinolic acid or pyrrothine), can be used by bacteria to produce thiomarinol. This and other studies on new thiomarinol secondary metabolites highlight the possibility of creating new hybrids by mutasynthesis [68,69] and, hence, to create new drugs with enhanced activity.

#### 3.2.8. *Cyclo*-Peptides

A *Pseudoalteromonas* sp. associated with the sponge *Halisarca ectofibrosa* has been isolated for its ability to inhibit *S. aureus*, *Micrococcus luteus*, *Bacillus subtilis*, *Escherichia coli* and *V. anguillarum* [70]*.* The resulting fermentation broth contains four *cyclo*-peptides: *cyclo*-(Phe-Pro-Leu-Pro), *cyclo*-(Leu-Pro)_2_, *cyclo*-(Phe-Leu)_2_ and *cyclo*-(Leu-Ile)_2_. Similar cyclic peptides are also produced by a strain of *Pseudomonas* [70]*.* Although the fermentation broth extracted with methanol inhibits several target bacteria, no inhibitory activity is recovered when *cyclo*-peptides are used alone; however, employing a solid phase synthesis method, the *cyclo*-(Phe-Pro-Leu-Pro) has been shown to exert a significant inhibitory activity against Gram-negative bacteria, notably against *Pseudomonas aeruginosa* and *Klebsellia oxytoca* [71]. The biosynthetic pathways involved in this biosynthesis of *cyclo*-peptides have not been investigated. The structural analogy with other tetrapeptides suggests that NRPS modules are at the origin of this peptide as in *Streptomyces* [72].

#### 3.2.9. Massetolides

**Figure 8:**
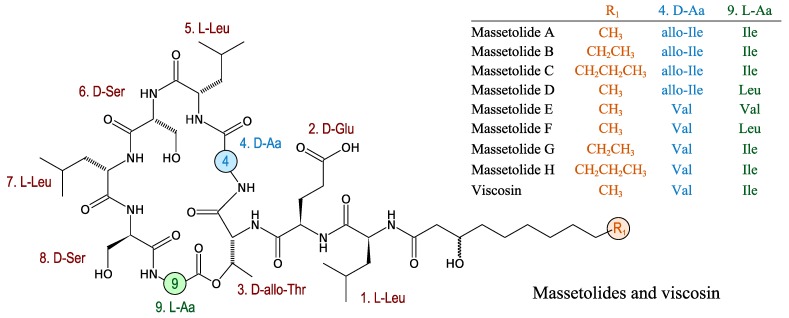
Structures of massetolides.

Massetolides are cyclic lipopeptides produced by other γ-Proteobacteria that have antimycobacterial activity. Massetolides A–D were first purified from a culture of a marine *Pseudomonas* MK90E85 isolated from an unidentified red alga collected in Masset Inlet, BC, Canada. Massetolides E–H and viscosin were first isolated from *Pseudomonas* MK91CC8 isolated from an unidentified tube worm that was collected near Moira Island, BC, Canada [73]. Massetolides are composed of nine amino acids with alternating d- or l-configurations and a variable length of fatty acid chains of variable length. Massetolides are cyclised through an ester linkage between the hydroxyl of the third amino acid residue and the carboxyl group of the last amino acid (Figure 8).

Massetolide A is synthesised by various strains of *Pseudomonas* from diverse environments. The biosynthesis of massetolides has been studied in *P. fluorescens* and has been shown to be governed by three NRPS modules designated MassA, MassB and MassC [74]. MassA (2089 amino acids) is composed of two modules whereas the MassB (4307 amino acids) and MassC (3775 amino acids) are respectively constituted of four and three modules. MassC also contains two TE domains. *In silico* analyses on A domain specificity are in accordance with a linear NRPS synthesis mechanism: The prediction of amino acids produced by MassA, B and C is consistent with the peptide part of massetolide A [74]. The two TE domains present at the end of MassC may work in tandem to enhance the rate of product release from the NRPS assembly line.

#### 3.2.10. Predicted NRPs

A collection of marine bacteria was created for its ability to inhibit some indicator species such as *B. subtilis*, *S. aureus*, *E. coli*, *Agrobacterium tumefaciens* and *Saccharomyces cerevisiae* [75]*.* Of these, one strain of *Pseudoalteromonas* named NJ631 inhibits the growth of all target strains and has been further analysed for its secondary metabolite biosynthetic genes [76]. Construction of a genomic library revealed a hybrid NRPS-PKS gene cluster in *Pseudoalteromonas* sp. NJ361. Bioinformatics analyses were used to predict the NRPS-PKS product. Four of the five A domains characterised (A2, A3, A4 and A5) have been successfully predicted for substrate specificity and were used to determine the composition of the nonribosomal peptide. It consists of one Glu or Gln, one Ser, one d-Ser and one Aeo (2-amino-9, 10-epoxy-8-oxodecanoic acid) [76]. A study on a NRP monomer has highlighted that certain residues generate specific biological activities of the peptide [77]. A search in the Norine database reveals that many NRPs containing Glu, Gln or d-Ser are known to display antibiotic activity. For instance, among the 199 NRPs containing Gln, 187 appear to exert antimicrobial activity. Furthermore, d-amino acids are frequently found in antibiotic NRPs [24]. d-Amino acid-based peptides seem to be particularly resistant to enzymatic degradation, which is of great interest for *in vivo* applications [78]. All these features suggest that this putative NRP has antibacterial activity. More structural information would allow the development of a purification protocol to confirm and study the antibiotic activity of this NRP from *Pseudoalteromonas*.

### 3.3. δ-Proteobacteria

#### 3.3.1. Myxovalargins

These molecules were discovered in a culture supernatant of a *Myxococcus fulvus* strain isolated from soil samples. This bacterium is also known for its halotolerance and ability to develop in marine seawater [79]*.* Myxovalargins are linear peptides ranging from 1500 to 1700 Da, containing Val, Arg and several unusual amino acids [80]. Being the most active molecule, myxovalargin A was further studied. It consists of 14 amino acids plus 3-methylbutyric acid and agmatine moieties; however its structure has not been fully elucidated [81].

Myxovalargin A is active against both Gram-positive and -negative bacteria and strongly represses protein synthesis in target bacteria at low concentrations, whereas its main effect at high concentrations is due to membrane permeabilisation [81].

To the best of our knowledge, no structure-activity relationship study has been undertaken for the myxovalargin family and their biosynthesis has not been investigated yet.

#### 3.3.2. Althiomycin

**Figure 9:**
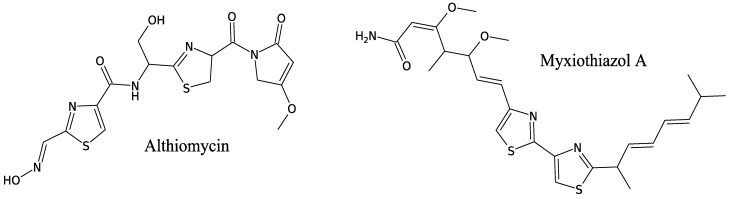
Antibiotic peptides from *Myxococcus fulvus*.

Althiomycin (Figure 9) is another peptide found in *M. fulvus* together with the antibiotic myxopyronins [82]. However, this molecule was originally described in the actinobacteria *Streptomyces althioticus* [83]*.*

Althiomycin is a pentapeptide containing two Gly, two Cys and one Ser and displays wide spectrum antibiotic activity against Gram-positive and -negative bacteria by inhibiting protein synthesis [84]. Its biosynthesis remains unknown in *M. fulvus*, but the production of althiomycin has been shown to involve a hybrid of NRPS and PKS systems in the closely related myxobacterium *Myxococcus xanthus* [85] and in the γ-Proteobacteria *Serratia marcescens* [86].

#### 3.3.3. Myxothiazols

Other antimicrobial polyketide-polypeptide hybrid metabolites discovered from *M. fulvus* are myxothiazols [87]. The structure of myxothiazol A is shown in Figure 9. Myxothiazols are obtained through the condensation of one 3-methylbutyrate, three acetates, two propionates and two Cys plus one undetermined terminal amino acid residue [88]. They exhibit anti-fungal activity [89,90] due to interferences with cytochrome b and inhibition of respiration. The synthesis of myxothiazols in *M. fulvus* has not been investigated. However, Perlova *et al*. describe the mechanism involving NRPS in another myxobacteria, *Stigmatella aurantiaca* [91].

To our knowledge, the production of myxovalargins, althiomycin or myxothiazols has not yet been demonstrated in marine species. Further experiments are required to determine if these metabolites can also be retrieved from marine *M. fulvus*. The search for bioactive compounds in marine myxobacteria can still be fruitful, as described below.

#### 3.3.4. Miuraenamides

**Figure 10:**
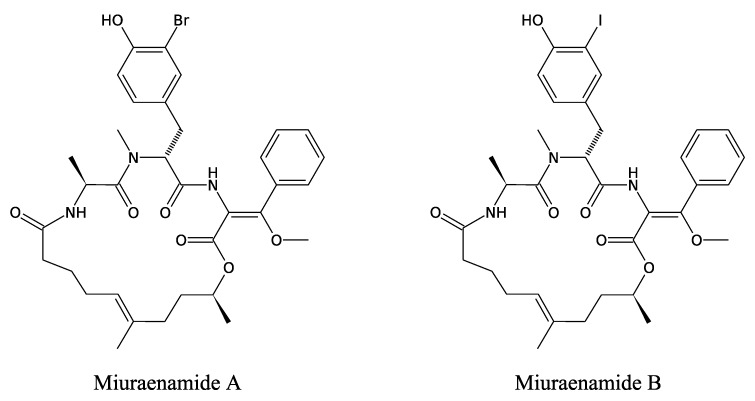
Antifungal peptides from *Paraliomyxa miuraensis*.

These are two antimicrobial compounds that have recently been identified from *Paraliomyxa miuraensis*, a new halophilic myxobacterium living on coastal soils [92], positioned close to the marine genera *Enhygromyxa* and *Plesiocystis* in a 16S rDNA phylogenetic tree. Miuraenamides A and B are cyclic depsipeptides that notably possess one halogenated *N*-methyl-Tyr, one dehydro-Phe and one Ala (Figure 10).

Miuraenamide A exerts significant antifungal activity against *Phytophthora capsici*, *Rhizopus oryzae* and *Candida rugosa*. The cyclic organisation in the molecules, as well as their lipophilicity and their phenol moiety, are involved in their activity [93]. The mechanism of synthesis of the miuraenamides has not yet been elucidated.

Beyond the recent discovery of marine myxobacteria and their potential as a source of antibiotics, the metabolome of other marine δ-Proteobacteria has been little investigated. However, most of the currently accepted marine genera of this class have been described only in the last decade. The recent descriptions particularly emphasise the sulphate- and sulphur-reducing ability of anaerobic marine/halophilic strains. To our knowledge, any putative production of antimicrobial molecules has yet to be assessed. In summary, even for myxobacteria, the chemical diversity of the δ*-*Proteobacteria class remains largely underexplored.

### 3.4. Molecules of Suspected Bacterial Origin

Since the late 20th century, the analysis of the metabolome of marine invertebrates has yielded numerous bioactive molecules. Marine eukaryotes frequently harbour bacteria. Since the 1990s, the true origin of secondary metabolites from marine invertebrates has been called in to question. In the next sections, only the antimicrobial peptides for which a proteobacterial origin is suspected or established will be reported. One of the best-documented cases involves the antifungal peptide theopalauamide.

#### 3.4.1. Theopalauamide

This molecule is a cyclic glycopeptide that displays numerous biological activities. Theopalauamide was originally reported from the lithistid sponge *Theonella swinhoei*, but it has since been attributed to a filamentous bacterial symbiont [94]. Schmidt *et al*. [95] discovered that theopalauamide is produced by a δ-Proteobacteria, *Candidatus* Entotheonalla palauensis, which appears to be closely related to the order Myxococcales. To date, attempts to cultivate this strain have remained unsuccessful. Theopalauamide is a rare example of an active molecule isolated from an animal and for which a bacterial origin has been fully established.

#### 3.4.2. Jaspamide and Related Peptides

**Figure 11:**
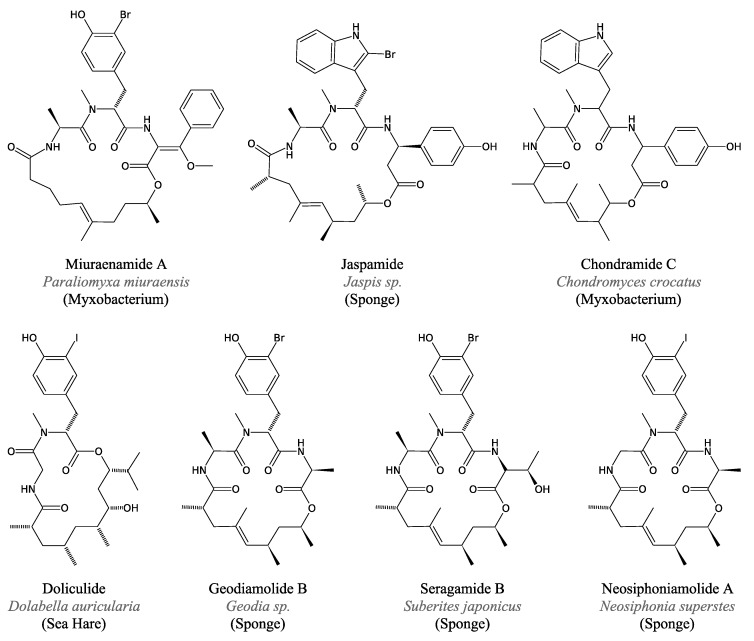
Jaspamide-related cyclic depsipeptides.

When they discovered miuraenamides from a new marine myxobacterium, Ojika *et al*. [93] also noticed the similarity between these molecules and other cyclic depsipeptides from marine organisms such as the geodiamolides [96], seragamides [97] and doliculide [98], which have all been isolated from invertebrates. The structures of these compounds are illustrated in Figure 11, together with those of the related jaspamide (or jasplakinolide) isolated from the sponge *Jaspis* sp. [99], neosiphoniamolide A from the sponge *Neosiphonia superstes* [100] and chondramides isolated from the soil-dwelling myxobacteria *Chondromyces crocatus* [101]. Considering their similarity and their invertebrate sources, many jaspamide-related peptides initially attributed to marine invertebrates may actually be produced by unknown marine myxobacteria [102].

Cyclic depsipeptides identified in marine invertebrates do indeed appear similar to other molecules from marine or terrestrial myxobacteria (Figure 11). As an example, the structures of both chondramide and jaspamide consist of three amino acids, *i.e*., one Ala, one *N*-methyl-Trp and one (α-methoxy) β-Tyr [103]. The only difference lies in the length and organisation of the polyketide chain (Figure 11).

Many biological activities have been reported for these jaspamide-related peptides. Their potential as antifungal or antibacterial agents has especially been highlighted since their discovery. One promising area of investigation involves their antitumour activity, which is very well documented for jaspamide, a microfilament inhibitor [104] (and references therein). Chondramide A induces the same effect as jaspamide on actin polymerisation [105]. Similar cytotoxic or anti-proliferative effects have also been demonstrated for miuraenamide A [106], doliculide [107], geodiamolide H [108] and seragamide A [97].

**Figure 12:**
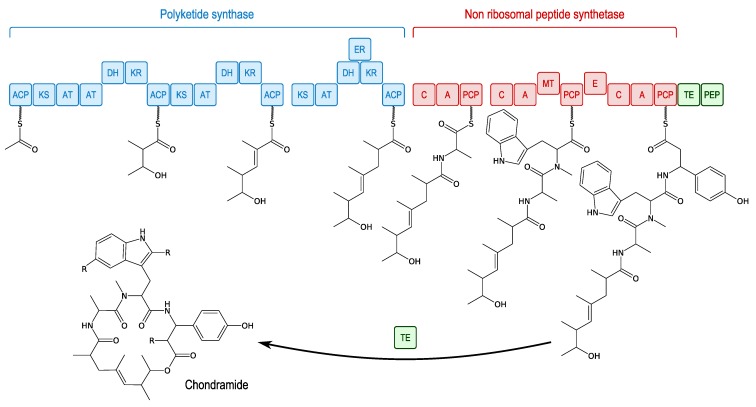
NRPS-PKS biosynthesis of the chondramides [103]. ACP: acyl carrier protein; KS: ketosynthase; AT: acyl transferase; DH: β-hydroxy dehydratase; KR: β-ketoacyl reductase; ER: enoyl reductase; C: condensation domain; A: adenylation domain; MT: methyltransferase domain; PCP: peptidyl carrier protein; E: epimerisation domain; TE: thioesterase domain; PEP: phosphoenolpyruvate domain.

A biosynthetic pathway has been described for chondramides from *Chondromyces crocatus* [103] (Figure 12). It consists of megasynthase systems, including both PKS and NRPS types (PKS-NRPS). Synthesis begins with the polyketide that is found in the cycle core of the chondramide; then Ala, Trp and finally β-Tyr are incorporated.

#### 3.4.3. Microsclerodermins and Pedeins

In 1994, Bewley *et al*. extracted and identified two new antifungal molecules, microsclerodermin A and B, from a lithistid sponge *Microscleroderma* sp. [109]. Because the antifungal activity was observed only in sponge-associated bacteria, the authors suggested that the sponge did not produce these molecules itself.

Microsclerodermins are cyclic peptides containing Gly as the only usual residue, *N*-methyl-Gly, 4-amino-3-hydroxybutyric acid, Trp-2-carboxylic acid, 3-hydroxy-4-amido-5-vinylpyrrolidone and 3-hydroxy-Asp. They strongly inhibit the growth of *C. albicans*.

The recent structural elucidation [110] of pedein A and B, other antifungal peptides from the myxobacteria *Chondromyces pediculatus*, brought new facts to light. These cyclic peptides are notably composed of (6-chloro)-Trp, Gly, and several unusual amino acids. Pedein A inhibits many yeasts and fungi, especially *C. albicans* and *Rhizopus arrhizus*.

**Figure 13:**
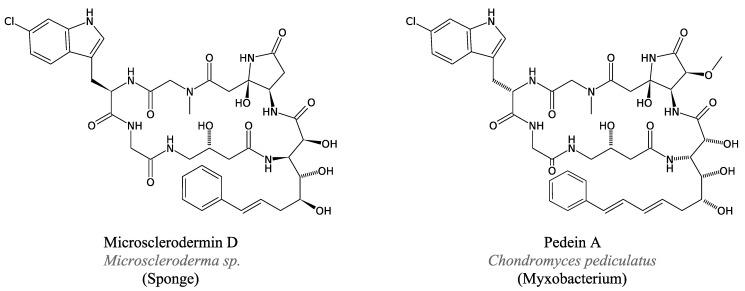
Cyclic peptides microsclerodermin D and pedein A.

A comparison of the structures of microscelordermin and pedein (Figure 13) suggests that *Microscleroderma* may not produce microsclerodermin and it is now thought that such peptides are produced by an unknown strain of marine proteobacteria [110].

The mechanism of synthesis of pedeins and microsclerodermins, possibly via NRPSs, is still undetermined.

#### 3.4.4. Saframycin and Related Peptides

**Figure 14:**
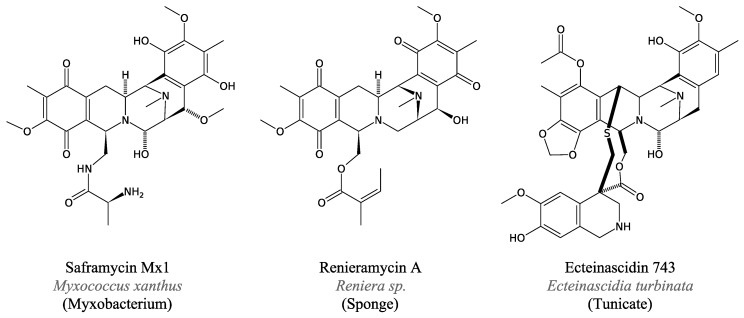
Saframycin-related peptides.

Saframycins and similar compounds from marine invertebrates present a similar case. Saframycin A was first discovered from *Streptomyces lavendulae* and displays antitumour and antibiotic activity [111]. Soon after, the antimicrobial renieramycin A (Figure 14) from the sponge *Reniera* sp. was reported and their resemblance with saframycins was noted [112].

The related saframycin Mx1 was first isolated from the myxobacteria *M. xanthus* and displays wide-spectrum antimicrobial activity, attributed to its efficient inhibition of DNA, RNA and protein synthesis *in vivo* [113]. It incorporates two Tyr, one Ala and one Gly via an NRPS pathway [114].

Ecteinascidin 743 was isolated [115] from a tunicate and displays antitumour properties [116,117]. This biological activity is at the origin of its use as an anti-cancer drug.

Even though the search for marine myxobacteria is still at an early stage, the numerous examples showing the impressive similarity between, on the one hand, sponge and ascidian antimicrobial peptides and, on the other hand, terrestrial or even marine myxobacterial peptides lead us to think that the discovery of new strains of marine myxobacteria will be a major area of investigation in the future. Such a source of new bioactive molecules should not be neglected. Several myxobacteria have been considered to be “unculturable”; the difficulty in their cultivation appears to be due to: (i) Their slow growth, preferentially in low-nutrient media [118]; and (ii) The absence of standardised media. Overcoming these issues will certainly lead to the discovery of new products and a better understanding of the marine invertebrate microbiota.

## 4. Conclusions

Antimicrobial peptides from marine Proteobacteria are generally obtained via nonribosomal pathways. They display potent antibacterial and/or antifungal activities. As a result, they may constitute useful tools to face the challenge of MDR strains. Marine NRPs from Proteobacteria exhibit a high level of structural diversity due to: (i) The presence of non-proteinogenic amino acid residues; (ii) The existence of multiple tailoring enzymes; and (iii) The possible association with PKS systems. Marine antibiotic NRPs are thus an important supply of molecules with great potential for biotechnological applications.

To screen for new NRPs, the genome-based approach is particularly pertinent, because this strategy allows the identification of silent genes and genes from uncultured bacteria. To date, the main characterised NRPS enzymes come from terrestrial organisms and particularly from Actinobacteria such as *Streptomyces* species. Nonetheless, the genomic approach based on NRPS homology cannot be a universal method to discover and analyse marine Proteobacteria NRPSs. The exclusive use of genomic strategies precludes discovering any non-databank-indexed structures. This highlights the need for a combination of genomic and microbiological strategies, such as complete genome analyses combined with natural NRPs purification and characterisation. However, this coupled approach requires culturable bacteria.

Despite the predominance of Proteobacteria in bacterioplankton, only a few NRPs have been isolated from this phylum when compared to the less abundant Cyanobacteria and Actinobacteria. The absence of standard cultivation protocols has limited investigations into their biochemistry. New cultivation methods have recently proven effective and can give access to the previously “unculturables”. This review has probably only just touched upon the tip of the iceberg. The structural diversity of antimicrobial NRPs (several unusual amino acids combined with diverse alkyl chains) described here is both an advantage and a disadvantage for use in a biotechnology context. Given the novel chemical structures, chemical synthesis must be defined. Access to this biotechnological source requires improving culture methods for Proteobacteria. With future advances in cultivation methods, we can reasonably expect an increase in the number of molecules isolated from marine Proteobacteria and the elucidation of new NRPS biosynthesis pathways, thus leading to new and efficient genome-based strategies.

## References

[1] Singh S.B., Barrett J.F. (2006). Empirical antibacterial drug discovery-foundation in natural products. Biochem. Pharmacol..

[2] Joint I., Mühling M., Querellou J. (2010). Culturing marine bacteria—An essential prerequisite for biodiscovery. Microb. Biotechnol..

[3] Fortman J.L., Sherman D.H. (2005). Utilizing the power of microbial genetics to bridge the gap between the promise and the application of marine natural products. Chembiochem.

[4] Giovannoni S., Stingl U. (2007). The importance of culturing bacterioplankton in the “omics” age. Nat. Rev. Microbiol..

[5] King G.M., Smith C.B., Tolar B., Hollibaugh J.T. (2012). Analysis of composition and structure of coastal to mesopelagic bacterioplankton communities in the northern gulf of Mexico. Front. Microbiol..

[6] Jamieson R.E., Rogers A.D., Billett D.S.M., Smale D.A., Pearce D.A. (2012). Patterns of marine bacterioplankton biodiversity in the surface waters of the Scotia Arc, Southern Ocean. FEMS Microbiol. Ecol..

[7] Lau S.C.K., Zhang R., Brodie E.L., Piceno Y.M., Andersen G., Liu W.-T. (2013). Biogeography of bacterioplankton in the tropical seawaters of Singapore. FEMS Microbiol. Ecol..

[8] Stackebrandt E., Fischer A., Roggentin T., Wehmeyer U., Bomar D., Smida J. (1988). A phylogenetic survey of budding, and/or prosthecate, non-phototrophic eubacteria: Membership of *Hyphomicrobium*, *Hyphomonas*, *Pedomicrobium*, *Filomicrobium*, *Caulobacter* and “Dichotomicrobium” to the alpha-subdivision of purple non-sulfur bacteria. Arch. Microbiol..

[9] Emerson D., Rentz J.A., Lilburn T.G., Davis R.E., Aldrich H., Chan C., Moyer C.L. (2007). A novel lineage of proteobacteria involved in formation of marine Fe-oxidizing microbial mat communities. PLoS One.

[10] Bhatnagar I., Se-Kwon K. (2010). Immense essence of excellence: Marine microbial bioactive compounds. Mar. Drugs.

[11] Gilbert J.A., Field D., Swift P., Newbold L., Oliver A., Smyth T., Somerfield P.J., Huse S., Joint I. (2009). The seasonal structure of microbial communities in the Western English Channel. Environ. Microbiol..

[12] Blunt J.W., Copp B.R., Munro M.H.G., Northcote P.T., Prinsep M.R. (2004). Marine natural products. Nat. Prod. Rep..

[13] Koglin A., Walsh C.T. (2009). Structural insights into nonribosomal peptide enzymatic assembly lines. Nat. Prod. Rep..

[14] Lipmann F., Gevers W., Kleinkauf H., Roskoski R. (1971). Polypeptide synthesis on protein templates: The enzymatic synthesis of gramicidin S and tyrocidine. Adv. Enzymol. Relat. Areas Mol. Biol..

[15] Lipmann F. (1973). Nonribosomal polypeptide synthesis on polyenzyme templates. Acc. Chem. Res..

[16] Kawakami T., Murakami H. (2012). Genetically encoded libraries of nonstandard peptides. J. Nucleic Acids.

[17] Schwarzer D., Finking R., Marahiel M.A. (2003). Nonribosomal peptides: From genes to products. Nat. Prod. Rep..

[18] Stachelhaus T., Mootz H.D., Bergendahl V., Marahiel M.A. (1998). Peptide bond formation in nonribosomal peptide biosynthesis catalytic role of the condensation domain. J. Biol. Chem..

[19] Hur G.H., Vickery C.R., Burkart M.D. (2012). Explorations of catalytic domains in non-ribosomal peptide synthetase enzymology. Nat. Prod. Rep..

[20] Weber T., Marahiel M.A. (2001). Exploring the domain structure of modular nonribosomal peptide synthetases. Structure.

[21] Schwarzer D., Marahiel M.A. (2001). Multimodular biocatalysts for natural product assembly. Naturwissenschaften.

[22] Lambalot R.H., Gehring A.M., Flugel R.S., Zuber P., LaCelle M., Marahiel M.A., Reid R., Khosla C., Walsh C.T. (1996). A new enzyme superfamily—The phosphopantetheinyl transferases. Chem. Biol..

[23] Nikolouli K., Mossialos D. (2012). Bioactive compounds synthesized by non-ribosomal peptide synthetases and type-I polyketide synthases discovered through genome-mining and metagenomics. Biotechnol. Lett..

[24] Caboche S., Pupin M., Leclère V., Fontaine A., Jacques P., Kucherov G. (2008). NORINE: A database of nonribosomal peptides. Nucleic Acids Res..

[25] Mootz H.D., Schwarzer D., Marahiel M.A. (2002). Ways of assembling complex natural products on modular nonribosomal peptide synthetases. Chembiochem.

[26] Blasiak L.C., Clardy J. (2010). Discovery of 3-formyl-tyrosine metabolites from *Pseudoalteromonas tunicata* through heterologous expression. J. Am. Chem. Soc..

[27] Bachmann B.O., Ravel J. (2009). Methods for *in silico* prediction of microbial polyketide and nonribosomal peptide biosynthetic pathways from DNA sequence data. Meth. Enzymol..

[28] Medema M.H., Blin K., Cimermancic P., de Jager V., Zakrzewski P., Fischbach M.A., Weber T., Takano E., Breitling R. (2011). AntiSMASH: Rapid identification, annotation and analysis of secondary metabolite biosynthesis gene clusters in bacterial and fungal genome sequences. Nucleic Acids Res..

[29] Ziemert N., Podell S., Penn K., Badger J.H., Allen E., Jensen P.R. (2012). The natural product domain seeker NaPDoS: A phylogeny based bioinformatic tool to classify secondary metabolite gene diversity. PLoS One.

[30] De Bruijn I., de Kock M.J.D., Yang M., de Waard P., van Beek T.A., Raaijmakers J.M. (2007). Genome-based discovery, structure prediction and functional analysis of cyclic lipopeptide antibiotics in *Pseudomonas* species. Mol. Microbiol..

[31] Li B., Walsh C.T. (2010). Identification of the gene cluster for the dithiolopyrrolone antibiotic holomycin in *Streptomyces clavuligerus*. Proc. Natl. Acad. Sci. USA.

[32] Park H.-M., Kim B.-G., Chang D., Malla S., Joo H.-S., Kim E.-J., Park S.-J., Sohng J.K., Kim P.I. (2013). Genome-based cryptic gene discovery and functional identification of NRPS siderophore peptide in *Streptomyces peucetius*. Appl. Microbiol. Biotechnol..

[33] Bumpus S.B., Evans B.S., Thomas P.M., Ntai I., Kelleher N.L. (2009). A proteomics approach to discovering natural products and their biosynthetic pathways. Nat. Biotechnol..

[34] Stein T., Vater J., Kruft V., Wittmann-Liebold B., Franke P., Panico M., Mc Dowell R., Morris H.R. (1994). Detection of 4′-phosphopantetheine at the thioester binding site for l-valine of gramicidin S synthetase 2. FEBS Lett..

[35] Wagner-Döbler I., Rheims H., Felske A., El-Ghezal A., Flade-Schröder D., Laatsch H., Lang S., Pukall R., Tindall B.J. (2004). *Oceanibulbus indolifex* gen. nov., sp. nov., a North Sea alphaproteobacterium that produces bioactive metabolites. Int. J. Syst. Evol. Microbiol..

[36] Milne P.J., Hunt A.L., Rostoll K., van der Walt J.J., Graz C.J. (1998). The biological activity of selected cyclic dipeptides. J. Pharm. Pharmacol..

[37] Slightom R.N., Buchan A. (2009). Surface colonization by marine roseobacters: Integrating genotype and phenotype. Appl. Environ. Microbiol..

[38] Cude W.N., Mooney J., Tavanaei A.A., Hadden M.K., Frank A.M., Gulvik C.A., May A.L., Buchan A. (2012). Production of the antimicrobial secondary metabolite indigoidine contributes to competitive surface colonization by the marine roseobacter *Phaeobacter* sp. strain Y4I. Appl. Environ. Microbiol..

[39] Oclarit J.M., Okada H., Ohta S., Kaminura K., Yamaoka Y., Iizuka T., Miyashiro S., Ikegami S. (1994). Anti-bacillus substance in the marine sponge, *Hyatella* species, produced by an associated *Vibrio* species bacterium. Microbios.

[40] Graff J.R., Forschner-Dancause S.R., Menden-Deuer S., Long R.A., Rowley D.C. (2013). *Vibrio cholerae* exploits sub-lethal concentrations of a competitor-produced antibiotic to avoid toxic interactions. Front. Microbiol..

[41] Wietz M., Mansson M., Gotfredsen C.H., Larsen T.O., Gram L. (2010). Antibacterial compounds from marine *Vibrionaceae* isolated on a global expedition. Mar. Drugs.

[42] Singh M.P., Mroczenski-Wildey M.J., Steinberg D.A., Andersen R.J., Maiese W.M., Greenstein M. (1997). Biological activity and mechanistic studies of andrimid. J. Antibiot..

[43] Jin M., Fischbach M.A., Clardy J. (2006). A biosynthetic gene cluster for the acetyl-CoA carboxylase inhibitor andrimid. J. Am. Chem. Soc..

[44] Fredenhagen A., Tamura S.Y., Kenny P.T.M., Komura H., Naya Y., Nakanishi K., Nishiyama K., Sugiura M., Kita H. (1987). Andrimid, a new peptide antibiotic produced by an intracellular bacterial symbiont isolated from a brown planthopper. J. Am. Chem. Soc..

[45] Freiberg C., Brunner N.A., Schiffer G., Lampe T., Pohlmann J., Brands M., Raabe M., Häbich D., Ziegelbauer K. (2004). Identification and characterization of the first class of potent bacterial acetyl-CoA carboxylase inhibitors with antibacterial activity. J. Biol. Chem..

[46] Pohlmann J., Lampe T., Shimada M., Nell P.G., Pernerstorfer J., Svenstrup N., Brunner N.A., Schiffer G., Freiberg C. (2005). Pyrrolidinedione derivatives as antibacterial agents with a novel mode of action. Bioorg. Med. Chem. Lett..

[47] Freiberg C., Pohlmann J., Nell P.G., Endermann R., Schuhmacher J., Newton B., Otteneder M., Lampe T., Häbich D., Ziegelbauer K. (2006). Novel bacterial acetyl coenzyme A carboxylase inhibitors with antibiotic efficacy *in vivo*. Antimicrob. Agents Chemother..

[48] Evans B.S., Chen Y., Metcalf W.W., Zhao H., Kelleher N.L. (2011). Directed evolution of the nonribosomal peptide synthetase AdmK generates new andrimid derivatives *in vivo*. Chem. Biol..

[49] Magarvey N.A., Fortin P.D., Thomas P.M., Kelleher N.L., Walsh C.T. (2008). Gatekeeping *versus* promiscuity in the early stages of the andrimid biosynthetic assembly line. ACS Chem. Biol..

[50] Gao J., Hamann M.T. (2011). Chemistry and biology of kahalalides. Chem. Rev..

[51] Hill R.T., Enticknap J., Rao K.V., Hamann M.T. (2004). Kahalalide-Producing Bacteria 2004. Eur. Pat. Appl..

[52] Shilabin A.G., Hamann M.T. (2011). *In vitro* and *in vivo* evaluation of select kahalalide F analogs with antitumor and antifungal activities. Bioorg. Med. Chem..

[53] Kenig M., Reading C. (1979). Holomycin and an antibiotic (MM 19290) related to tunicamycin, metabolites of *Streptomyces clavuligerus*. J. Antibiot..

[54] Oliva B., O’Neill A., Wilson J.M., O’Hanlon P.J., Chopra I. (2001). Antimicrobial properties and mode of action of the pyrrothine holomycin. Antimicrob. Agents Chemother..

[55] Khachatourians G.G., Tipper D.J. (1974). *In vivo* effect of thiolutin on cell growth and macromolecular synthesis in *Escherichia coli*. Antimicrob. Agents Chemother..

[56] Urbanczyk H., Ast J.C., Dunlap P.V. (2011). Phylogeny, genomics, and symbiosis of *Photobacterium*. FEMS Microbiol. Rev..

[57] Hazen T.H., Pan L., Gu J.-D., Sobecky P.A. (2010). The contribution of mobile genetic elements to the evolution and ecology of *Vibrios*. FEMS Microbiol. Ecol..

[58] Oku N., Kawabata K., Adachi K., Katsuta A., Shizuri Y. (2008). Unnarmicins A and C, new antibacterial depsipeptides produced by marine bacterium *Photobacterium* sp. MBIC06485. J. Antibiot..

[59] Yoshikawa K., Adachi K., Nishida F., Mochida K. (2003). Planar structure and antibacterial activity of korormicin derivatives isolated from *Pseudoalteromonas* sp. F-420. J. Antibiot..

[60] Adachi K., Kawabata Y., Kasai H., Katsuta M., Shizuri Y. (2007). Novel Ngercheumicin or Its Salt Useful for Treating Infection Caused by *Pseudovibrio denitrificans*. Japanese Patent.

[61] Mansson M., Gram L., Larsen T.O. (2011). Production of bioactive secondary metabolites by marine Vibrionaceae. Mar. Drugs.

[62] Finch R.G., Pritchard D.I., Bycroft B.W., Williams P., Stewart G.S. (1998). Quorum sensing: A novel target for anti-infective therapy. J. Antimicrob. Chemother..

[63] Wise M.P., Williams D.W., Lewis M.A.O., Frost P.J. (2010). Macrolides and community-acquired pneumonia: Is quorum sensing the key?. Crit. Care.

[64] Defoirdt T., Boon N., Bossier P. (2010). Can bacteria evolve resistance to quorum sensing disruption?. PLoS Pathog..

[65] Shiozawa H., Kagasaki T., Kinoshita T., Haruyama H., Domon H., Utsui Y., Kodama K., Takahashi S. (1993). Thiomarinol, a new hybrid antimicrobial antibiotic produced by a marine bacterium: fermentation, isolation, structure, and antimicrobial activity. J. Antibiot..

[66] Shiozawa H., Kagasaki T., Torikata A., Tanaka N., Fujimoto K., Hata T., Furukawa Y., Takahashi S. (1995). Thiomarinols B and C, new antimicrobial antibiotics produced by a marine bacterium. J. Antibiot..

[67] Shiozawa H., Shimada A., Takahashi S. (1997). Thiomarinols D, E, F and G, new hybrid antimicrobial antibiotics produced by a marine bacterium; isolation, structure, and antimicrobial activity. J. Antibiot..

[68] Fukuda D., Haines A.S., Song Z., Murphy A.C., Hothersall J., Stephens E.R., Gurney R., Cox R.J., Crosby J., Willis C.L. (2011). A natural plasmid uniquely encodes two biosynthetic pathways creating a potent anti-MRSA antibiotic. PLoS One.

[69] Murphy A.C., Fukuda D., Song Z., Hothersall J., Cox R.J., Willis C.L., Thomas C.M., Simpson T.J. (2011). Engineered thiomarinol antibiotics active against MRSA are generated by mutagenesis and mutasynthesis of *Pseudoalteromonas* SANK73390. Angew. Chem. Int. Ed. Engl..

[70] Rungprom W., Siwu E.R.O., Lambert L.K., Dechsakulwatana C., Barden M.C., Kokpol U., Blanchfield J.T., Kita M., Garson M.J. (2008). Cyclic tetrapeptides from marine bacteria associated with the seaweed *Diginea* sp. and the sponge *Halisarca ectofibrosa*. Tetrahedron.

[71] Dahiya R., Gautam H. (2011). Toward the synthesis and biological screening of a cyclotetrapeptide from marine bacteria. Mar. Drugs.

[72] Binz T.M., Maffioli S.I., Sosio M., Donadio S., Muller R. (2010). Insights into an unusual nonribosomal peptide synthetase biosynthesis. J. Biol. Chem..

[73] Gerard J.M. (1992). Antibiotic Secondary Metabolites of Bacteria Isolated from the Marine Environment. Ph.D. Thesis.

[74] De Bruijn I., de Kock M.J.D., de Waard P., van Beek T.A., Raaijmakers J.M. (2008). Massetolide A biosynthesis in *Pseudomonas fluorescens*. J. Bacteriol..

[75] Zheng L., Han X., Chen H., Lin W., Yan X. (2005). Marine bacteria associated with marine macroorganisms: The potential antimicrobial resources. Ann. microbiol..

[76] Zhu P., Zheng Y., You Y., Yan X., Shao J. (2009). Sequencing and modular analysis of the hybrid non-ribosomal peptide synthase-polyketide synthase gene cluster from the marine sponge *Hymeniacidon perleve*-associated bacterium *Pseudoalteromonas* sp. strain NJ631. Can. J. Microbiol..

[77] Caboche S., Leclère V., Pupin M., Kucherov G., Jacques P. (2010). Diversity of monomers in nonribosomal peptides: Towards the prediction of origin and biological activity. J. Bacteriol..

[78] Wade D., Boman A., Wåhlin B., Drain C.M., Andreu D., Boman H.G., Merrifield R.B. (1990). All-d amino acid-containing channel-forming antibiotic peptides. Proc. Natl. Acad. Sci. USA.

[79] Li Z.-F., Li X., Liu H., Liu X., Han K., Wu Z.-H., Hu W., Li F., Li Y.-Z. (2011). Genome sequence of the halotolerant marine bacterium *Myxococcus fulvus* HW-1. J. Bacteriol..

[80] Irschik H., Gerth K., Kemmer T., Steinmetz H., Reichenbach H. (1983). The myxovalargins, new peptide antibiotics from *Myxococcus fulvus* (Myxobacterales). I. Cultivation, isolation, and some chemical and biological properties. J. Antibiot..

[81] Irschik H., Reichenbach H. (1985). The mechanism of action of myxovalargin A, a peptide antibiotic from *Myxococcus fulvus*. J. Antibiot..

[82] Irschik H., Gerth K., Höfle G., Kohl W., Reichenbach H. (1983). The myxopyronins, new inhibitors of bacterial RNA synthesis from *Myxococcus fulvus* (Myxobacterales). J. Antibiot..

[83] Yamaguchi H., Nakayama Y., Takeda K., Tawara K., Maeda K., Takeuchi T., Umezawa H. (1957). A new antibiotic, althiomycin. J. Antibiot..

[84] Fujimoto H., Kinoshita T., Suzuki H., Umezawa H. (1970). Studies on the mode of action of althiomycin. J. Antibiot..

[85] Cortina N.S., Revermann O., Krug D., Müller R. (2011). Identification and characterization of the althiomycin biosynthetic gene cluster in *Myxococcus xanthus* DK897. Chembiochem.

[86] Gerc A.J., Song L., Challis G.L., Stanley-Wall N.R., Coulthurst S.J. (2012). The insect pathogen *Serratia marcescens* Db10 uses a hybrid non-ribosomal peptide synthetase-polyketide synthase to produce the antibiotic althiomycin. PLoS One.

[87] Trowitzsch W., Reifenstahl G., Wray V., Gerth K. (1980). Myxothiazol, an antibiotic from *Myxococcus fulvus* (Myxobacterales). II. Structure elucidation. J. Antibiot..

[88] Silakowski B., Schairer H.U., Ehret H., Kunze B., Weinig S., Nordsiek G., Brandt P., Blöcker H., Höfle G., Beyer S. (1999). New lessons for combinatorial biosynthesis from myxobacteria. The myxothiazol biosynthetic gene cluster of *Stigmatella aurantiaca* DW4/3-1. J. Biol. Chem..

[89] Thierbach G., Reichenbach H. (1981). Myxothiazol, a new antibiotic interfering with respiration. Antimicrob. Agents Chemother..

[90] Ahn J.-W., Jang K.H., Yang H.-C., Oh K.-B., Lee H.-S., Shin J. (2007). Bithiazole metabolites from the myxobacterium *Myxococcus fulvus*. Chem. Pharm. Bull..

[91] Perlova O., Fu J., Kuhlmann S., Krug D., Stewart A.F., Zhang Y., Müller R. (2006). Reconstitution of the myxothiazol biosynthetic gene cluster by Red/ET recombination and heterologous expression in *Myxococcus xanthus*. Appl. Environ. Microbiol..

[92] Iizuka T., Fudou R., Jojima Y., Ogawa S., Yamanaka S., Inukai Y., Ojika M. (2006). Miuraenamides A and B, novel antimicrobial cyclic depsipeptides from a new slightly halophilic myxobacterium: Taxonomy, production, and biological properties. J. Antibiot..

[93] Ojika M., Inukai Y., Kito Y., Hirata M., Iizuka T., Fudou R. (2008). Miuraenamides: Antimicrobial cyclic depsipeptides isolated from a rare and slightly halophilic myxobacterium. Chem. Asian J..

[94] Bewley C.A., Faulkner D.J. (1998). Lithistid sponges: Star performers or hosts to the stars. Angew. Chem. Int. Ed..

[95] Schmidt E.W., Obraztsova A.Y., Davidson S.K., Faulkner D.J., Haygood M.G. (2000). Identification of the antifungal peptide-containing symbiont of the marine sponge *Theonella swinhoei* as a novel δ-Proteobacterium, “*Candidatus* Entotheonella palauensis”. Mar. Biol..

[96] Chan W.R., Tinto W.F., Manchand P.S., Todaro L.J. (1987). Stereostructures of geodiamolides A and B, novel cyclodepsipeptides from the marine sponge *Geodia* sp.. J. Org. Chem..

[97] Tanaka C., Tanaka J., Bolland R.F., Marriott G., Higa T. (2006). Seragamides A–F, new actin-targeting depsipeptides from the sponge *Suberites japonicus* Thiele. Tetrahedron.

[98] Ishiwata H., Nemoto T., Ojika M., Yamada K. (1994). Isolation and stereostructure of doliculide, a cytotoxic cyclodepsipeptide from the Japanese sea hare *Dolabella auricularia*. J. Org. Chem..

[99] Zabriskie T.M., Klocke J.A., Ireland C.M., Marcus A.H., Molinski T.F., Faulkner D.J., Xu C., Clardy J. (1986). Jaspamide, a modified peptide from a *Jaspis* sponge, with insecticidal and antifungal activity. J. Am. Chem. Soc..

[100] D’Auria M.V., Gomez Paloma L., Minale L., Zampella A., Debitus C., Perez J. (1995). Neosiphoniamolide A, a novel cyclodepsipeptide, with antifungal activity from the marine sponge *Neosiphonia superstes*. J. Nat. Prod..

[101] Kunze B., Jansen R., Sasse F., Höfle G., Reichenbach H. (1995). Chondramides A approximately D, new antifungal and cytostatic depsipeptides from *Chondromyces crocatus* (Myxobacteria). Production, physico-chemical and biological properties. J. Antibiot..

[102] Weissman K.J., Müller R. (2009). A brief tour of myxobacterial secondary metabolism. Bioorg. Med. Chem..

[103] Rachid S., Krug D., Kunze B., Kochems I., Scharfe M., Zabriskie T.M., Blöcker H., Müller R. (2006). Molecular and biochemical studies of chondramide formation-highly cytotoxic natural products from *Chondromyces crocatus* Cm c5. Chem. Biol..

[104] Ebada S.S., Wray V., de Voogd N.J., Deng Z., Lin W., Proksch P. (2009). Two new jaspamide derivatives from the marine sponge *Jaspis splendens*. Mar. Drugs.

[105] Sasse F., Kunze B., Gronewold T.M., Reichenbach H. (1998). The chondramides: Cytostatic agents from myxobacteria acting on the actin cytoskeleton. J. Natl. Cancer Inst..

[106] Sumiya E., Shimogawa H., Sasaki H., Tsutsumi M., Yoshita K., Ojika M., Suenaga K., Uesugi M. (2011). Cell-morphology profiling of a natural product library identifies bisebromoamide and miuraenamide A as actin filament stabilizers. ACS Chem. Biol..

[107] Matcha K., Madduri A.V.R., Roy S., Ziegler S., Waldmann H., Hirsch A.K.H., Minnaard A.J. (2012). Total synthesis of (−)-doliculide, structure-activity relationship studies and its binding to F-actin. Chembiochem.

[108] Freitas V.M., Rangel M., Bisson L.F., Jaeger R.G., Machado-Santelli G.M. (2008). The geodiamolide H, derived from Brazilian sponge *Geodia corticostylifera*, regulates actin cytoskeleton, migration and invasion of breast cancer cells cultured in three-dimensional environment. J. Cell. Physiol..

[109] Bewley C.A., Debitus C., Faulkner D.J. (1994). Microsclerodermins A and B antifungal cyclic peptides from the lithistid sponge *Microscleroderma* sp.. J. Am. Chem. Soc..

[110] Kunze B., Böhlendorf B., Reichenbach H., Höfle G. (2008). Pedein A and B: Production, isolation, structure elucidation and biological properties of new antifungal cyclopeptides from *Chondromyces pediculatus* (Myxobacteria). J. Antibiot..

[111] Arai T., Takahashi K., Nakahara S., Kubo A. (1980). The structure of a novel antitumor antibiotic, saframycin A. Experientia.

[112] Frincke J.M., Faulkner D.J. (1982). Antimicrobial metabolites of the sponge *Reniera* sp.. J. Am. Chem. Soc..

[113] Irschik H., Trowitzsch-Kienast W., Gerth K., Höfle G., Reichenbach H. (1988). Saframycin Mx1, a new natural saframycin isolated from a myxobacterium. J. Antibiot..

[114] Pospiech A., Cluzel B., Bietenhader J., Schupp T. (1995). A new *Myxococcus xanthus* gene cluster for the biosynthesis of the antibiotic saframycin Mx1 encoding a peptide synthetase. Microbiology.

[115] Rinehart K.L., Holt T.G., Fregeau N.L., Stroh J.G., Keifer P.A., Sun F., Li L.H., Martin D.G. (1990). Ecteinascidins 729, 743, 745, 759A, 759B, and 770: Potent antitumor agents from the Caribbean tunicate *Ecteinascidia turbinata*. J. Org. Chem..

[116] Grohar P.J., Griffin L.B., Yeung C., Chen Q.-R., Pommier Y., Khanna C., Khan J., Helman L.J. (2011). Ecteinascidin 743 interferes with the activity of EWS-FLI1 in Ewing sarcoma cells. Neoplasia.

[117] Erba E., Bergamaschi D., Bassano L., Damia G., Ronzoni S., Faircloth G.T., D’Incalci M. (2001). Ecteinascidin-743 (ET-743), a natural marine compound, with a unique mechanism of action. Eur. J. Cancer.

[118] Garcia R.O., Krug D., Müller R. (2009). Discovering natural products from myxobacteria with emphasis on rare producer strains in combination with improved analytical methods. Meth. Enzymol..

